# Non-genotoxic carcinogens (TPA and mezerein) activate tumourous transformation through miR let-7-mediated Hmga2 expression in Bhas42 cells

**DOI:** 10.1093/eep/dvaf005

**Published:** 2025-03-03

**Authors:** Moon Yi Ko, Euijun Min, Minjeong Kim, Heejin Park, Sumi Jang, Younhee Kim, Byoung-Seok Lee, Sung-Ae Hyun, Minhan Ka

**Affiliations:** Department of Advanced Toxicology Research, Korea Institute of Toxicology, Daejeon 34114, Republic of Korea; Department of Biochemistry, Chungnam National University, Daejeon 34134, Republic of Korea; Department of Advanced Toxicology Research, Korea Institute of Toxicology, Daejeon 34114, Republic of Korea; Department of Biochemistry, Chungnam National University, Daejeon 34134, Republic of Korea; Department of Advanced Toxicology Research, Korea Institute of Toxicology, Daejeon 34114, Republic of Korea; Department of Advanced Toxicology Research, Korea Institute of Toxicology, Daejeon 34114, Republic of Korea; Department of Advanced Toxicology Research, Korea Institute of Toxicology, Daejeon 34114, Republic of Korea; Department of Advanced Toxicology Research, Korea Institute of Toxicology, Daejeon 34114, Republic of Korea; Department of Advanced Toxicology Research, Korea Institute of Toxicology, Daejeon 34114, Republic of Korea; Department of Advanced Toxicology Research, Korea Institute of Toxicology, Daejeon 34114, Republic of Korea; Department of Advanced Toxicology Research, Korea Institute of Toxicology, Daejeon 34114, Republic of Korea; Human and Environmental Toxicology, University of Science and Technology, Daejeon 34114, Republic of Korea

**Keywords:** non-genotoxic carcinogens, TPA, mezerein, miR let-7, Hmga2, Ezh2

## Abstract

A Bhas42 cell transformation assay is a method used to detect the tumour-promoting activities of chemicals. However, the mechanisms underlying tumour transformations mediated by non-genotoxic carcinogens (NGCs) are poorly understood. This study aimed to examine the correlation between 12-*O*-tetradecanoylphorbol 13-acetate (TPA) or mezerein and the initiation of tumourous transformations by epigenetic regulation in Bhas42 cells. We found that TPA and mezerein prompted tumourous transformations by stimulating cell proliferation and migration in Bhas42 cells. Furthermore, we observed alterations in the expression levels of 134 genes, with 87 genes being upregulated and 47 genes being downregulated, following exposure to either TPA or mezerein. Among the differentially regulated genes, we identified 17 upregulated genes and 8 downregulated genes corresponding to differentially expressed genes in TNM [primary tumour (T), regional nodes (N), and metastasis (M)]. Importantly, we found that TPA and mezerein triggered the expression of Hmga2 and Ezh2 by loss of miRNA let-7 (miR let-7) in Bhas42 cells. Finally, the microRNA (miRNA) mimic of let-7 prevented the TPA- and mezerein-induced activation of Hmga2 and Ezh2 in Bhas42 cells. Our findings reveal a connection between tumourous transformations and the epigenetic regulator miR let-7 in NGCs, such as TPA and mezerein in Bhas42 cells. This highlights miR let-7 as a promising therapeutic target for mitigating tumourous transformations induced by NGCs.

## Introduction

Cancer, a complex disease characterized by uncontrolled cell growth and proliferation, remains a significant public health concern worldwide [1]. Carcinogens have been classified into two main categories: genotoxic and non-genotoxic types, providing a logical basis for cancer risk assessment. A ‘genotoxic carcinogen’ refers to a substance that induces cancer by directly damaging the genetic material of target cells. In contrast, a ‘non-genotoxic carcinogen (NGCs)’ describes a substance that promotes cancer through secondary mechanisms unrelated to direct genetic damage [2]. NGCs initiate or promote carcinogenesis through mechanisms independent of direct DNA interactions. These mechanisms involve alterations in cell signalling pathways, disruption of cellular homeostasis, modulation of gene expression patterns, or promotion of chronic inflammation [3]. The cell transformation assay (CTA) is a vital tool for assessing the carcinogenic potential of substances, particularly NGCs [4]. CTA using Bhas42 cells is a well-established method for evaluating the carcinogenic potential of various chemicals and compounds [5]. Bhas42 cells, derived from rat liver epithelial cells, are particularly sensitive to chemically induced transformations, making them valuable models for studying carcinogenesis [6]. In a previous study, Bhas42 cells were exposed to a test substance over a defined period, allowing for the assessment of morphological and phenotypic changes associated with cell transformation [7]. These changes include altered cell morphology, increased proliferation rate, loss of contact inhibition, and acquisition of anchorage-independent growth, which are characteristic features of transformed cells [8].

NGCs are linked to epigenetic modifications during cancer development [9]. They play a role in DNA methylation, histone methylation, and histone acetylation, influencing tumour initiation and progression. In addition to alterations in DNA and histones, epigenetic regulation also involves the distribution and function of RNA-binding proteins and noncoding RNAs (ncRNAs) [10]. Among ncRNAs, microRNAs (miRNAs), long-noncoding RNAs (lncRNAs), and circular RNAs have been extensively investigated for their distinct roles in cancer progression [11]. miRNAs are small ncRNAs that play crucial roles in the regulation of gene expression at the post-transcriptional level [7]. Among these, miR let-7 has emerged as a key player in controlling carcinogenesis [12]. Let-7 was initially discovered as a developmental regulator in *Caenorhabditis elegans* [13], but its dysregulation has been implicated in various human cancers [14]. As a tumour suppressor miRNA, miR let-7 is often downregulated in cancer cells, leading to the depression of its target genes involved in cell proliferation, apoptosis, differentiation, and metastasis [15, 16]. Conversely, certain oncogenes negatively regulate miR let-7 expression, creating a feedback loop that perpetuates carcinogenic processes [17]. Through its ability to target multiple genes within oncogenic signaling pathways, miR let-7 profoundly affects tumour initiation, progression, and metastasis [18, 19]. Emerging evidence suggests that miR let-7 may modulate the tumour microenvironment and influence immune responses, angiogenesis, and drug resistance [20–23].

In this study, we consistently observed that NGCs such as 12-*O*-tetradecanoylphorbol 13-acetate (TPA)- or mezerein-induced tumourous transformation by enhancing cell proliferation and migration in Bhas42 cells. These findings shed light on the molecular mechanisms underlying NGC-induced carcinogenesis and offer valuable insights into the complex interplay between environmental exposure and cancer development.

## Results

### TPA or mezerein activates transformed foci formation and proliferation in Bhas42 cells

Initially, we investigated whether exposure to TPA or mezerein can induce the formation of transformed foci using the CTA assay in Bhas42 cells.

**Figure 1.**
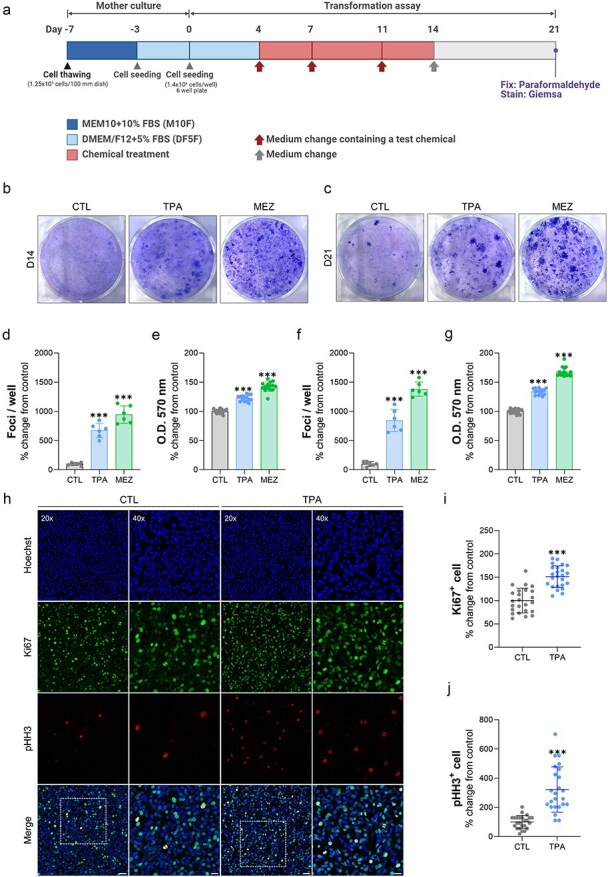
TPA and mezerein promotes the formation of foci and proliferation of Bhas42 cells. (a) The experimental procedures of the Bhas 42 cell transforming assay. Representative images of transformed foci induced by exposure to TPA or mezerein on Day 14 (b) and Day 21 (c). Quantification of transformed foci (d) and absorbance (e) shown in (b). Quantification of transformed foci (f) and absorbance (g) shown in (c). The graphs show an analysis of the number of transformed foci in treated compared to control groups. Data are presented as the mean ± standard error of the mean (*n* = 6). ^***^P < .001 vs. of control. TPA promotes cell proliferation on Day 21 in Bhas42 cells. (h) Representative images show cell proliferation in Bhas42 cell; cell nuclei marker: Hoechst, cell proliferation marker: Ki67 in green and pHH3 in red. Scale bar = 40 µm, 20 µm. Quantification of Ki67 (i) and pHH3 (j) normalized to total number of the DAPI. Data are presented as the mean ± standard error of the mean (*n* = 34). ^***^P < .001 vs. of control.

Bhas42 cells underwent two subcultures in an M10F medium for 4 days each, after which they were harvested. Subsequently, the cells were seeded onto a 6-well plate and cultured for 4 days. After this initial culture period, the cells were exposed to either 50 ng/ml TPA or 15 ng/ml mezerein for 10 days. Following exposure, the cells were cultured in a plain medium for either 1 or 7 days before Giemsa staining (Fig. 1a). We observed a significant increase in the number of transformed foci in the Bhas42 cells cultured for 14 days or 21 days when induced by either TPA or mezerein (Fig. 1b–d).

To investigate the effects of TPA on cell proliferation, Bhas42 cells cultured for 14 days or 21 days were seeded on coverslips in 24-well plates and cultured for 24 h. We assessed actively proliferating cells and mitotic phase cells in TPA-exposed Bhas42 cells and controls by immunostaining with Ki67 and phospho-histone H3 antibodies. We found that the number of Ki67+ cells increased by 49% in the TPA-exposed Bhas42 cells compared to that in the controls (Fig. 1e, f). Furthermore, we observed a 240% increase in the number of phospho-histone H3+ cells in the TPA-exposed Bhas42 cells compared to that in the controls (Fig. 1e, g). These findings suggest that TPA has a significant impact on transformed foci formation and proliferation in Bhas42 cells, indicating its potential role in the activation of tumour progression in Bhas42 cells exposed to NGCs.

### TPA activates cell migration and invasion in Bhas42 cells

**Figure 2.**
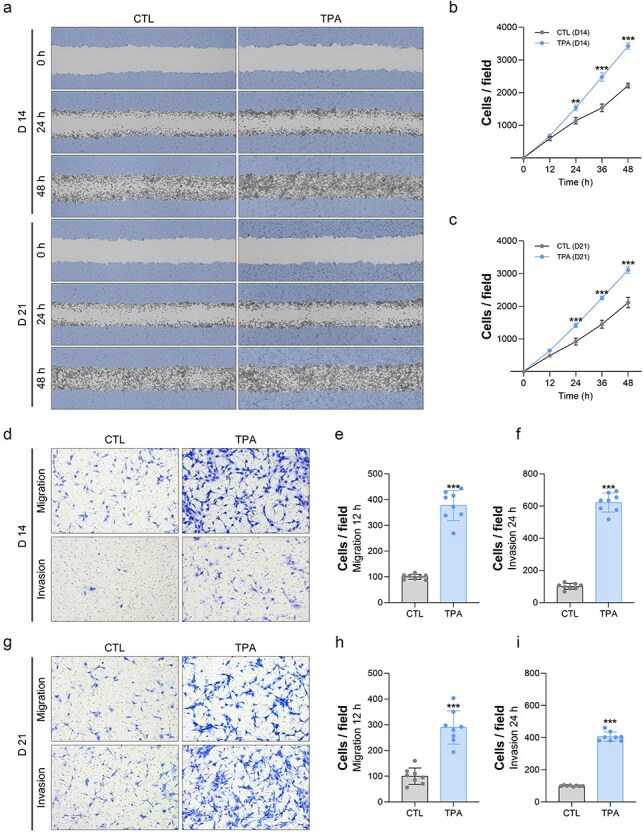
TPA enhances the migration and invasion of Bhas42 cells. (a) Representative images from the wound healing assay depict the migration of cells in the presence of TPA at various time intervals (0, 24, and 48 h) on Days 14 and 21 for Bhas42 cells. The data show quantification of the migration rate vs. the control on Days 14 (b) and 21 (c). Data are presented as the mean ± standard error of the mean (*n* = 4). ^**^P < .01 and ^***^P < .001 vs. of control. (d) The image provided depicts the stained cell invasion ability using Giemsa on Day 14 following treatment with TPA. Quantification of migration (e) and invasion (f) are shown in Figure (d). (g) The image provided depicts the stained cell invasion ability using Giemsa on Day 21 following treatment with TPA. Quantification of migration (h) and invasion (i) are shown in Figure (g). Data are presented as the mean ± standard error of the mean (*n* = 8). ^***^P < .001 vs. of control.

We investigated whether exposure to TPA induces cell migration in Bhas42 cells using a wound-healing assay. For this, Bhas42 cells cultured for 14 days or 21 days were seeded in Ibidi culture-insert 2-well dishes and cultured for 24 or 48 h. We found that the number of migrated cells increased in a time-dependent manner in the TPA-exposed Bhas42 cells cultured for 14 days compared to that in the control cells (Fig. 2a, b). Furthermore, we found that the number of migrated cells increased in a time-dependent manner in the TPA-exposed Bhas42 cells cultured for 21 days compared with that in the controls (Fig. 2a, c).

Next, to confirm whether exposure to TPA can induce cell migration and invasion in Bhas42 cells, we performed transwell *in vitro* cell migration and invasion assays. Bhas42 cells cultured for 14 days or 21 days were seeded in transwell plates and cultured for 12 or 24 h. In Bhas42 cells cultured for 14 days, TPA exposure resulted in a 277% increase in the number of migrated cells compared to that in the controls (Fig. 2d, e). Additionally, the number of invading cells in TPA-exposed Bhas42 cells cultured for D14 increased by 520% compared to that in the controls (Fig. 2d, f). Consistently, we found that in Bhas42 cells cultured for 21 days, TPA exposure resulted in a 189% increase in the number of migrated cells and a 306% increase in the number of invading cells compared to the controls (Fig. 2g–i). These findings suggested that TPA significantly activated the migration and invasion of Bhas42 cells.

### TPA or mezerein changes the gene expression profile in Bhas42 cells during CTA

**Figure 3.**
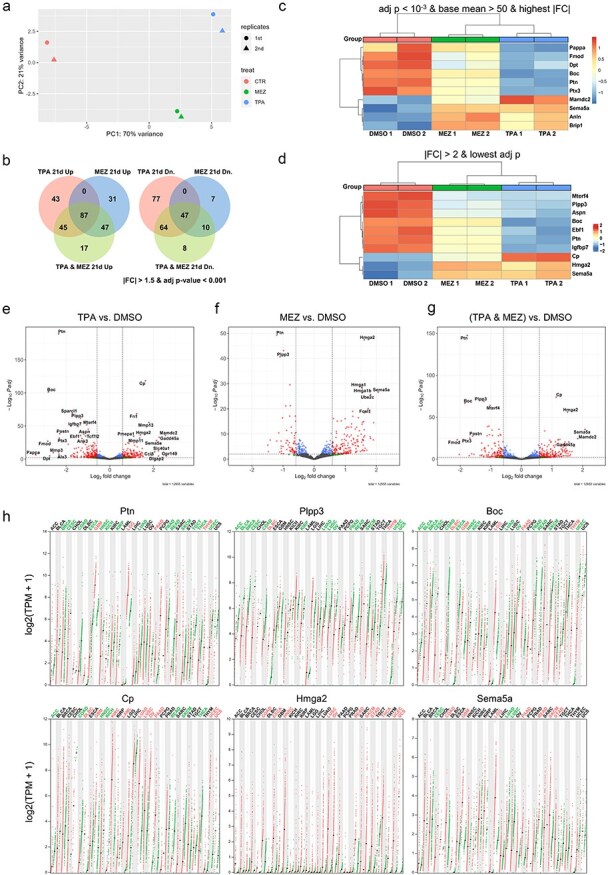
DEGs in TPA or mezerein exposed Bhas42 according to the 21-day CTA protocol. (a) PCA plot of DMSO, TPA, and mezerein group. (b) Comparison among the three types of DEGs. Heat map of Top-10 DEGs in TPA–mezerein with the highest|FC| within base mean >100 (C) or lowest adj-*P* within| FC|> 2 after Ashr shrinkage (d). (e–g) Volcano plots of TPA, mezerein, and TPA–mezerein. (f) Expression levels of selected DEGs in human cancers. Each dot represents each sample value, and the black bars are median values. In coloured cancer types, the target gene passed the DEGs cut-off (|log2FC|> 1 and *q* < .01; red: high in cancer, green: high in normal.

We investigated whether exposure to TPA or mezerein can affect the mRNA transcriptome pattern in Bhas42 cells. The transcriptomic effects of TPA and mezerein on Day 21 of the CTA were assessed using QuantSeq-3 mRNA-seq. According to a principal component analysis (PCA), replicates clustered well within each chemical group, and the distance between TPA and mezerein was smaller than that between the control and either chemical (Fig. 3a). Differentially expressed genes (DEGs) were identified through three comparisons: TPA vs. DMSO, mezerein vs. DMSO, and the averages of TPA and mezerein vs. DMSO (TNM vs. DMSO). We found that all the common DEGs between the TPA and mezerein were also included in the TNM DEGs, comprising 87 upregulated and 47 downregulated genes (Fig. 3b). Additionally, some genes that were not DEGs in either TPA or mezerein individually became DEGs in the grouped comparison (17 upregulated and 8 downregulated) (Fig. 3b). Among the prominent DEGs in the TNM group (Fig. 3c, d), six were selected based on the |ashr FC|> 2 criteria and the lowest adjusted *P*-value, and their expression in human cancer was investigated (Fig. 3e). The top three downregulated DEGs (Ptn, Plpp3, and Boc) (Fig. 3d) were found to be underexpressed in many tumour types (tumour names in green), with a threshold of|log2FC|> 1 and *q* < 0.01. However, significant overexpression was detected in a few tumour types (red) (Fig. 3e). The expression of the two upregulated DEGs (Cp and Sema5a) was dependent on the cancer type. However, Hmga2 showed no underexpression in any cancer type in either the GEPIA or Oncopression datasets and was upregulated in 16 out of 19 tumour types (Fig. 3e).

### TPA or mezerein time-dependently activates Hmga2 expression in Bhas42 cells during CTA

**Figure 4.**
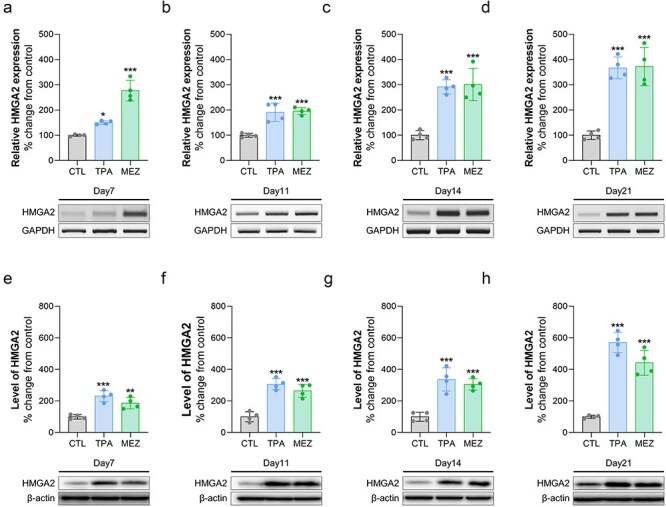
TPA and mezerein increases the expression of the HMGA2 gene and protein. (a–d) The HMGA gene expression levels were altered on Days 7, 11, 14, and 21 after treatment with TPA and mezerein in Bhas42 cells. (e–h) The levels of HMGA protein were changed on Days 7, 11, 14, and 21 following treatments with TPA and mezerein in Bhas42 cells. Data are presented as the mean ± standard error of the mean (*n* = 4). *P < .05, ^**^P < .01 and ^***^P < .001 vs. of control.

We investigated the activation of Hmga2 expression in Bhas42 cells exposed to TPA or mezerein during a CTA. First, Bhas42 cells were exposed to TPA or mezerein for specified time intervals (Day 4, Day 7, and Day 11) during the CTA and assessed for changes in mRNA transcription levels of Hmga2 at specified time intervals (Day 7, Day 11, Day 14, and Day 21). We found that the transcription level of Hmga2 increased in a time-dependent manner in TPA-exposed Bhas42 cells during the CTA (Fig. 4a–d). Furthermore, we witnessed time-dependent activation of the transcription levels of Hmga2 in mezerein-exposed Bhas42 cells during the CTA (Fig. 4a–d). Next, we assessed the changes in the protein levels of Hmga2 at specific time intervals (Day 7, Day 11, Day 14, and Day 21). Consistently, we found that the protein levels of Hmga2 increased in a time-dependent manner in TPA-exposed Bhas42 cells during the CTA (Fig. 4e–h). We also found that the level of Hmga2 exhibited a significant and time-dependent increase in mezerein-exposed Bhas42 cells during the CTA (Fig. 4e–h). These findings suggest that TPA or mezerein activates the expression levels of Hmga2 in Bhas42 cells during CTAs, indicating a molecular target for tumour progression activation in Bhas42 cells exposed to NGCs.

### TPA or mezerein activates the transcription levels of cancer-related genes in Bhas42 cells during CTA

**Figure 5.**
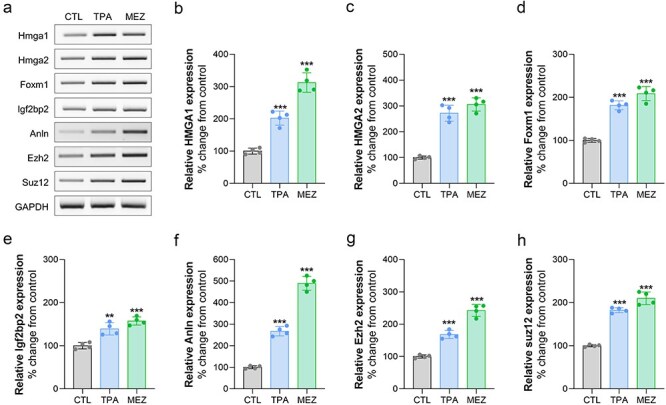
TPA and mezerein increases the expression of cancer-related genes. (a) Representative image of expression changes in cancer-related genes in Bhas42 cells following treatment with TPA and mezerein. (b–h) Quantification of protein expression shown in Figure (a). Data are presented as the mean ± standard error of the mean (*n* = 4). ^**^P < .01 and ^***^P < .001 vs. of control.

We investigated the potential impact of TPA or mezerein exposure on cancer-related genes in Bhas42 cells. During the CTA, Bhas42 cells were exposed to TPA or mezerein, and the mRNA levels of cancer-related genes were assessed using RT-PCR. We found that the transcription levels of Hmga1 and Hmga2 increased by 87% and 169%, respectively, in TPA-exposed Bhas42 cells compared to the controls (Fig. 5a–c). Additionally, we found that the mRNA levels of Hmga1 and Hmga2 increased by 197% and 210%, respectively, in mezerein-treated Bhas42 cells compared to the controls (Fig. 5a–c). We also found that the mRNA levels of Foxm1 were increased by 84% and 95% in TPA- and mezerein-treated Bhas42 cells, respectively, compared to that in the controls (Fig. 5a, d). Moreover, the levels of Igf2bp2 were slightly increased by 37% and 50% in TPA- and mezerein-exposed Bhas42 cells, respectively, compared to that in the controls (Fig. 5a, e). Next, we assessed the transcription levels of Anln, Ezh2, and Suz12 in TPA- and mezerein-treated Bhas42 cells. We found that the transcription levels of Anln, Ezh2, and Suz12 increased by 175%, 77%, and 84%, respectively, in TPA-exposed Bhas42 cells compared to that in the controls (Fig. 5a, f–h). Additionally, we found that the mRNA levels of Anln, Ezh2, and Suz12 increased by 387%, 137%, and 93%, respectively, in mezerein-treated Bhas42 cells compared to that in the controls (Fig. 5a, f–h). These findings suggest that TPA or mezerein activates the expression levels of cancer-related genes in Bhas42 cells during CTAs.

### miR Let-7 restores cell proliferation by down-regulating the expression of Hmga2 and Ezh2

**Figure 6.**
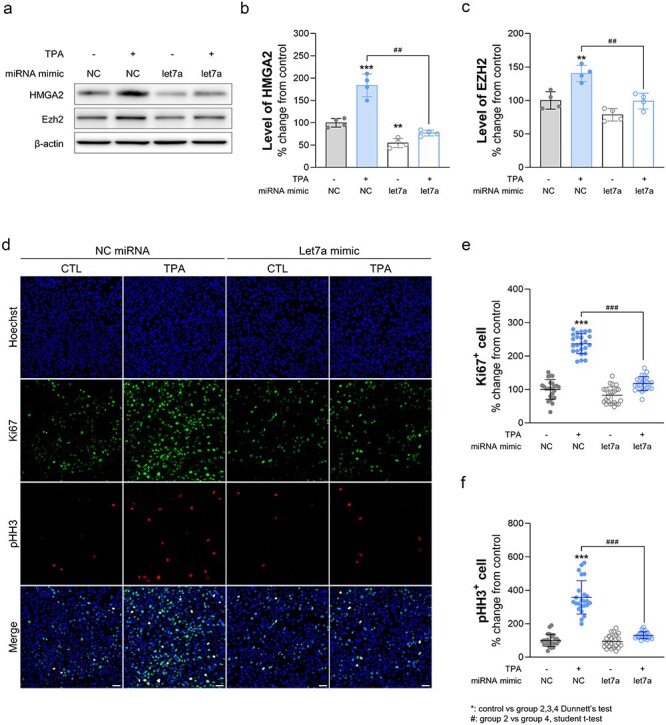
TPA induces cancer progression through miR let-7-mediated Ezh2 expression. (a) HMGA2 and Ezh2 protein expression changes with let-7a miRNA treatment in TPA-exposed Bhas42 cells. (b, c) Quantification of protein expression shown in Figure (a). Data are presented as the mean ± standard error of the mean (*n* = 4). (d) Representative images show cell proliferation with let-7a miRNA treatment in TPA-exposed Bhas42 cells; cell nuclei marker: DAPI in blue, cell proliferation marker: Ki67 in green and pHH3 in red. Scale bar = 40 µm, 20 µm. Quantification of Ki67 (e) and pHH3 (f) normalized to total number of the DAPI. Data are presented as the mean ± standard error of the mean (*n* = 24). Statistical comparisons are performed as follows; *multiple comparisons between group 1 and groups 2, 3, and 4, ^#^: *t*-test between group 2 and group 4.

Based on previous studies, miR let-7 functions as a tumour suppressor by targeting and downregulating various oncogenes involved in essential cellular processes such as proliferation, differentiation, and apoptosis [17]. Additionally, it specifically represses the HMGA2 oncogene [24]. With this knowledge in mind, we investigated whether miR let-7 suppresses tumour progression in TPA-treated Bhas42 cells. First, Bhas42 cells were subcultured in M10F medium for 4 days, after which they were harvested. Subsequently, the cells were seeded onto a 6-well plate and cultured for 4 days. After this initial culture period, the Bhas42 cells were transfected with the let-7 mimic and then exposed to TPA for 10 days. Following exposure, the cells were cultured in a plain medium for an additional 7 days. We then assessed the levels of Hmga2 and Ezh2 in the Bhas42 cell lysates. Consistently, we found that the level of Hmga2 increased by 84% in the TPA-exposed Bhas42 cells (Fig. 6a, 6B). However, the miR let-7 mimic reduced Hmga2 expression in the TPA-treated Bhas42 cells (Fig. 6a, b). Similarly, we found that the level of Ezh2 increased by 40% in TPA-treated Bhas42 cells (Fig. 6a, c), but the miR let-7 mimic reduced Ezh2 expression in these cells (Fig. 6a, c). Next, we assessed actively proliferating and mitotic phase cells by immunostaining with Ki67 and phospho-histone H3 antibodies. We found that the number of Ki67+ cells increased by 137% in TPA-exposed Bhas42 cells compared to that in the controls (Fig. 6d, e). However, the miR let-7 mimic reduced the number of Ki67+ cells in the TPA-treated Bhas42 cells (Fig. 6d, e). Furthermore, we observed a 157% increase in the number of phospho-histone H3+ cells in TPA-exposed Bhas42 cells compared to that in the controls (Fig. 6d, f). Again, the miR let-7 mimic reduced the number of phospho-histone H3+ cells in the TPA-treated Bhas42 cells (Fig. 6d, f). These findings suggest a critical role for miR let-7 in inactivating Hmga2 and Ezh2 in TPA-treated Bhas42 cells during CTAs.

## Discussion

**Figure 7.**
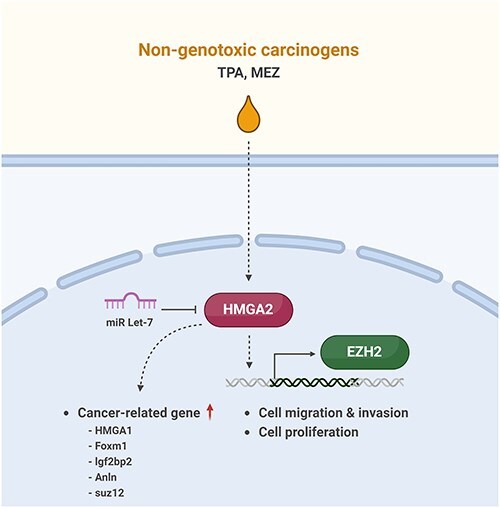
A schematic diagram illustrating the progression of cancer caused by non-genotoxic carcinogens. TPA and mezerein leads to an increase in the expression of HMGA2 and EZH2, subsequently facilitating cell migration, invasion, and proliferation. TPA and mezerein are also capable of enhancing the expression of genes that are linked to the development and progression of cancer.

TPA and mezerein are both compounds that are classified as NGCs [25, 26]. Unlike genotoxic carcinogens, which cause direct DNA damage leading to mutations and cancer, nongenotoxic carcinogens promote cancer through mechanisms that do not involve direct DNA damage [27, 28]. Instead, these agents may influence cellular processes such as proliferation, apoptosis, and differentiation [2, 29]. Despite the high accuracy of Bhas 42 CTA in identifying tumour-promoting agents, as highlighted by the OECD study (ENV/JM/MONO(2016)1), the assay’s lengthy duration and the complexity of the molecular processes involved have hindered its full regulatory acceptance [30]. Our study demonstrates that TPA and mezerein significantly induce transformed foci formation and proliferation in Bhas42 cells through the epigenetic regulation of Hmga2 and Ezh2, suggesting their role in tumour progression (Fig. 7). It has been previously reported that TPA is used as a tumour-promoting agent in rodent skin carcinogenesis studies and is associated with increased cell proliferation in malignant cells from various tumours, including melanoma, breast cancer, and oral cancer [31–33]. Our findings also demonstrated that exposure to TPA resulted in a substantial increase in transformed foci and cell proliferation, as evidenced by the increased numbers of Ki67+ and phospho-histone H3+ cells. These markers indicate enhanced cell proliferation and mitotic activity, respectively, highlighting the potential of TPA to drive tumorigenic processes in Bhas42 cells. Moreover, TPA exposure markedly enhances cell migration and invasion, which are crucial steps in cancer metastasis. Wound healing and Transwell assays revealed that TPA not only increased the number of migrated cells but also significantly boosted the invasion capacity of Bhas42 cells. Consistently, TPA-induced cell migration and invasion via the PKCδ-mtROS-HSP60 MAPK-AP1 pathway [34]. TPA stimulates breast cancer cell motility by modulating the expression and activity of S100A14 in a KLF4-dependent manner [35]. It also induces glioblastoma cell invasion and migration by activating PKCα/ERK/NF-κB-dependent MMP-9 expression [36]. Overall, these findings highlight the aggressive nature of TPA-induced cellular changes that promote a more invasive phenotype in Bhas42 cells.

NGCs, such as TPA and mezerein, are known to promote tumourigenesis by altering the epigenetic landscape, including DNA methylation, histone modifications, and ncRNA regulation [10, 37, 38]. Among key epigenetic regulators, Ezh2, a histone methyltransferase and a central component of the Polycomb Repressive Complex 2 (PRC2), has been shown to drive tumour progression through H3K27 trimethylation (H3K27me3)-mediated transcriptional repression of tumour suppressor genes [39–41]. In our study, we observed a significant upregulation of Ezh2 following TPA and mezerein exposure, suggesting a potential epigenetic mechanism contributing to tumour transformation in Bhas42 cells. Additionally, the tumour-suppressive role of miR let-7 is well-documented across multiple cancer types [24]. Let-7 downregulation is often associated with the depression of oncogenes such as Hmga2, a key factor involved in chromatin remodelling and cell proliferation [42]. Moreover, let-7 is known to regulate DNA methyltransferases and histone-modifying enzymes, further underscoring its role in epigenetic regulation [43]. Based on our findings, we propose that NGCs may exert their tumour-promoting effects by disrupting miRNA-mediated epigenetic regulation, leading to the dysregulation of oncogenic pathways.

Our transcriptomic analysis using QuantSeq-3ʹ mRNA-seq revealed that TPA and mezerein exposure alters the gene expression profile in Bhas42 cells. The PCA showed distinct clustering of the TPA and mezerein groups from the control, with overlapping DEGs between the TPA and mezerein treatments. Notably, several DEGs were exclusively identified in the combined TPA and mezerein group (TNM), indicating a synergistic effect on gene expression. Among these DEGs, key genes, such as Hmga2, were consistently upregulated, indicating their role in tumourigenesis. Moreover, Hmga2 is abnormally regulated in various human cancers including lung, breast, and ovarian cancers, and its increased expression is associated with a high risk of cancer progression [44–46]. Our investigation into the time-dependent activation of Hmga2 expression during the CTA revealed that both TPA and mezerein significantly upregulate Hmga2 at the mRNA and protein levels over time. This consistent increase suggests that Hmga2 may serve as a crucial molecular target in TPA- and mezerein-induced tumour progression in Bhas42 cells.

Further analysis of cancer-related genes showed that TPA and mezerein exposure upregulates several oncogenes, including Hmga1, Foxm1, Igf2bp2, Anln, Ezh2, and Suz12. The upregulation of these genes highlights the extensive impact of TPA and mezerein on promoting oncogenic pathways, reinforcing their role in enhancing the tumourigenic potential of Bhas42 cells. Hmga1 is overexpressed in several types of cancer, including breast, colorectal, and pancreatic cancers [47]. Elevated levels of Hmga1 are associated with enhanced tumourigenicity, metastasis, and poor prognosis [34]. Hmga1 remodels chromatin to drive developmental transcriptional networks in cancer [48]. Hmga1 protein interacts with nucleosomal DNA and modifies nucleosome architecture by altering the periodicity of DNA wrapping. Through its role in chromatin remodelling, Hmga1 contributes to epigenetic regulation, influencing gene expression and cellular functions. These structural changes can affect DNA accessibility, thereby playing a crucial role in transcriptional regulation and cancer progression [49]. Foxm1, an oncogenic transcription factor crucial for cancer initiation, progression, and drug resistance, is significantly upregulated in various cancers, including breast cancer [50, 51]. Hmga2 transactivates Foxm1 [52]. The binding of Hmga2 to a proximal region of the Igf2bp2 oncogene results in the activation of its transcription [53]. Igf2bp2 modulates cancer cell proliferation, migration, invasion, metastasis, and apoptosis by regulating the transcription of miRNAs, lncRNAs, and other m6A-related genes [54]. Hmga2 can directly regulate Anln expression [55]. Anln is highly expressed in many types of site-specific cancer tumours, including those of the brain, lungs, pancreas, and bone marrow [56]. Ezh2, histone methyltransferase and key component of Prc2, catalyses H3K27me3 to regulate gene expression epigenetically. It functions as both a transcriptional suppressor and activator, depending on context. Ezh2 plays a crucial role in cancer by promoting cell survival, proliferation, and invasion [57]. Higher expression levels of Ezh2 and Suz12 are strongly correlated with tumour progression and overall survival [58].

Through its ability to target multiple genes within oncogenic signaling pathways, miR let-7 profoundly affects tumour initiation, progression, and metastasis. Moreover, emerging evidence suggests that miR let-7 may modulate the tumour microenvironment, influencing immune responses, angiogenesis, and drug resistance [24, 59–61]. Importantly, our study identified the tumour-suppressive role of miR let-7 in counteracting the oncogenic effects of TPA. Transfection with the let-7 mimic effectively reduced the expression of Hmga2 and Ezh2, leading to a significant decrease in cell proliferation and mitotic activity in TPA-treated Bhas42 cells. This finding aligns with previous studies indicating that miR let-7 targets and downregulates oncogenes such as Hmga2, thereby suppressing tumourigenesis. The ability of miR let-7 to mitigate the effects of TPA underscores its potential therapeutic value in targeting TPA-induced tumour progression.

In conclusion, our study provides strong evidence that TPA and mezerein induce epigenetic regulation in Bhas42 cells, promoting transformed foci formation, proliferation, migration, and invasion. These alterations are closely associated with DNA methylation, histone modifications, and noncoding RNA regulation, which collectively contribute to cancer progression. Notably, the upregulation of oncogenes, particularly Hmga2, alongside the tumour-suppressive function of miR let-7, highlights key molecular targets and potential therapeutic approaches for counteracting TPA-driven tumour development. Further studies are required to elucidate the intricate epigenetic pathways involved and to explore the therapeutic potential of miR let-7 in cancer treatment.

## Materials and methods

### Bhas42 cells culture and chemical exposure

Bhas 42 cells were purchased from Japanese Collection of Research Bioresources Cell Bank (JCRB0149, Japan). TPA (524400, Calbiochem) and mezerein (SC-202707, Santa Cruz Biotechnology) were dissolved in dimethyl sulfoxide (DMSO) (D2650, Sigma-Aldrich). Bhas 42 cells were maintained and exposed to chemicals according to the 21-day CTA protocol (Fig. 1a) as outlined in the Organization for Economic Cooperation and Development (OECD) Guidance Document No. 231 [62]. Briefly, cells were plated on Day 0 in Dulbecco’s Modified Eagle Medium/Nutrient Mixture F-12 (DMEM/F12) containing 5% Fetal Bovine Serum (FBS) (DF5F, 10565-018, Gibco; 1.4 × 10^4^ cells/well for 6-well plates or 400 cells/well for 96-well plates) and were exposed to each chemical from Day 4 to Day 14 (a total of 10 days; 50 ng/ml TPA, 0.5 ng/ml mezerein, and 0.1% DMSO as solvent control). After 7 additional days of culture without chemical treatment Day 14 to Day 21, cells were fixed and Giemsa stained (48900, Sigma-Aldrich). During the 21 days of culture, portions of the culture plates were collected on Day 7, Day 11, Day14, and Day 21 with Trizol (15596 018, Ambion) and Radio-Immunoprecipitation Assay (RIPA) buffer (89901, Thermo Scientific) to obtain RNA and protein samples, respectively. Concurrently, cells for the growth assay were seeded (400 cells/well, 96-well plates) and exposed to each chemical from Day 4 to Day 7. On Day 7, cell growth was measured by MTT assay (G4000, Promega) or crystal violet extraction (C0775, Sigma-Aldrich). For Let-7a mimic transfection, let-7a-5p miRCURY LNA miRNA Mimic (hsa-let-7a-5p, which also targets mmu-let7a-5p; 339173, Qiagen) and negative control (YM00479902, Qiagen) were transfected with Lipofectamine 3000 (L3000015, Invitrogen™) 6 h before the chemical treatment (15 pmol/well for 6-well plates, 2.5 pmol/well for 24-well plates, and 0.5 pmol/well for 96-well plates).

### Wound closure and transwell assay

On Day 14 and Day 21 of CTA, subsets of Bhas 42 cells were harvested with 0.25% Trypsin-EDTA (25200 056, Gibco). For wound closure assay, the cells were seeded into 6-well plates (1× 10^6^ cells/well). After 12 h of culture, scratch wounds were made by a pipette tip and media changed (DMEM/F12 containing 2.5% FBS). The wounds were captured every 12 h and binary-converted images were analysed by ImageJ. For trans-well assays, harvested cells were resuspended with 0% FBS media and seeded into inserts (5 × 10^4^ cells/well. 3422, Costar). Before that, half of the inserts were overnight coated with Matrigel for invasion assay; the others uncoated were used for migration assay. The bottom wells were filled with DF5F and cultured for 12 h. After fixation with 4% PFA (PC2031, Biosesang) and 0.1% crystal violet staining, five random fields in each membrane were captured and cells were counted.

### Immunostaining

Immunofluorescence of Bhas42 cells was carried out following established methods as described [63, 64]. Cultured Bhas42 cells were fixed using 4% paraformaldehyde for 10 min and permeabilized with 0.5% Triton X-100 for 10 min at room temperature. Subsequently, the fixed cells were blocked with 5% Normal Goat Serum in PBS buffer and incubated overnight at 4°C with primary antibodies against Ki67 (11-5698-82, Thermo Scientific) and p-Histone H3 (#9701, Cell Signaling). Following the primary antibody incubation, the cells were probed using appropriate Alexa Fluor-conjugated secondary antibodies (A-11 006 or A-11 012, Invitrogen) for 1 h at room temperature. Images were captured using an Olympus FV3000 microscope and Olympus software (Olympus Life Science).

### Reverse transcription polymerase chain reaction

Reverse transcription PCR analysis was conducted following established procedures as outlined [65, 66]. RNA was extracted from cultured cells using Trizol reagent (Thermo Fisher Scientific Inc.). cDNA was synthesized from 1 μg of total RNA using oligo-dT and random hexamers with the Verso cDNA synthesis kit (Thermo Fisher Scientific Inc.). PCR was performed using 1 μg of RNA to generate cDNA with Maxime™ RT PreMix (25081, iNtRON Biotechnology). Following primer sequences were used: Hmga1 forward 5′-caagcagcctccggtgagtc-3′ and reverse 5′-gggtctgccccttggtttcc-3′; Hmga2 forward 5′-gacccaggaagcagcagcaa-3′ and reverse 5′-ctctgcggactcttgcgagg-3′; Foxm1 forward 5′-gcccgtcatagcaagcgagt-3′ and reverse 5′-aaaggcaacagcccttcccc-3′; Igf2bp2 forward 5′-acactgcgcggaatggtgaa-3′ and reverse 5′-tcctcctcctgttccccagc-3′; Anln forward 5′-cgcatgcgaagagaggcaga-3′ and reverse 5′-ggtgcaggcatcggagaaca-3′; Ezh2 forward 5′-ccatgctacctggctgtccg-3′ and reverse 5′-acatcagacggtgccagcag-3′; Suz12 forward 5′-cagctcctgttgccaagcct-3′ and reverse 5′-cagtgcaggtcgtctctggc-3′; *Gapdh* forward 5′-tgtggatggcccctctggaa-3′ and reverse 5′-ttggcaggtttctccaggcg-3′. All primers were initially tested for their specificity by running RT-PCR samples on an agarose gel.

### Western blotting

Western blotting analysis was conducted following established procedures as outlined [67, 68]. Total protein was extracted from Bhas42 cells using RIPA lysis buffer (89900, Thermo Scientific). For each sample, 20 μg of protein were separated on a 6% or 10% SDS gel and transferred onto a PVDF transfer membrane (88520, Thermo Scientific). Subsequently, the PVDF membrane was blocked with 5% BSA in TBS-T buffer and incubated overnight at 4°C with primary antibodies against Ezh2 (#5246, Cell Signaling), Hmga2 (#5269, Cell Signaling), and β-actin (SC-517582, Santa Cruz). The PVDF membrane was then probed with appropriate HRP-conjugated secondary antibodies (65-6120, 62-6520, and 31470, Invitrogen) for 1 h at room temperature. Protein bands were detected using ECL reagents (34075, Thermo Scientific) and the ImageQuant LAS 500 system (GE Healthcare). Quantification of protein bands was performed using ImageJ software, and the results were represented as relative intensity versus control.

### Quantseq-3ʹmRNA-seq and differentially expressed genes analysis

Quantseq-3ʹ mRNA-seq (https://doi.org/10.1038/nmeth.f.376) was performed using mRNA obtained from D21 samples treated with TPA, mezerein, and DMSO, with two replicates for each condition. DEGs were analysed using the DEseq2 package [69] in R. Shrunken log2 fold-changes (log2FC) were calculated using the Ashr method [70] for DEGs filtering and further analysis. Three types of contrasts were applied: TPA vs. DMSO, mezerein vs. DMSO, and the average of TPA and mezerein (TNM) vs. DMSO. In each contrast, DEGs were selected with a threshold of|log2FC| > log2(1.5) and an adjusted *P*-value < .01.

### Gene enrichment analysis

Functional enrichment was analysed and visualized using the ClusterProfiler 4.0 package in R [71]. Over-representation analysis (ORA) was conducted with up- or down-regulated DEGs, utilizing the gene-set library in Enrichr [72] for analysing ChIP targets. Gene set enrichment analysis (GSEA) was performed with total genes arranged in decreasing order of Ashr shrunken log2FC. After the analysis, similar gene ontology (GO) terms were removed using the GOSemSim package [73].

### Comparison with microarray data

Some microarray probes can bind to multiple genes or splicing variants. To simplify the comparison among the DEGs in previous microarrays [74, 75] and our RNA-Seq data, every gene detected by significantly different probes was considered a DEG. For the 48-h DEGs, data from GSE133279 in Gene Expression Omnibus (GEO) were analysed using GEO2R after selecting TPA and DMSO groups. For the ORA background, total genes detectable by the given microarrays were extracted from platform information in GEO (GPL1261 for 1 h to 8 days, GPL21163 for 48 h). Before comparing DEGs, gene symbols were re-annotated with up-to-date gene IDs and symbols using AnnotationDbi with org.Mm.eg.db in R. Missing IDs and symbols were manually checked and updated with replaced values if possible. Venn diagrams and UpSet plots were drawn using the VennDiagram and UpsetR packages in R, respectively. Intersection elements were also obtained using these packages.

### Statistical analysis

Statistical analysis was conducted using GraphPad Prism software (GraphPad Software, Inc.). All data are presented as mean ± SEM. Statistical significance between two groups was determined using a Student’s *t*-test, while multiple comparisons were evaluated using a one-way ANOVA followed by the Bonferroni post hoc test. A significance level of *P* < .05 was considered for determining statistical significance.

## Data Availability

The authors declare that the data supporting the findings of this study are available within the paper. If any raw data files be needed in another format, they are available from the corresponding author upon reasonable request.
